# The Impact of Macronutrients on Retinal Microvasculature among Singapore Pregnant Women during the Mid-Late Gestation

**DOI:** 10.1371/journal.pone.0160704

**Published:** 2016-08-10

**Authors:** Ling-Jun Li, Peng Guan Ong, Marjorelee T. Colega, Chad Yixian Han, Ling Wei Chen, Ryan Man Eyn Kidd, Ecosse Lamoureux, Peter Gluckman, Kenneth Kwek, Yap Seng Chong, Seang Mei Saw, Keith M. Godfrey, Tien Yin Wong, Mary Chong Foong-Fong, GUSTO study group

**Affiliations:** 1 Singapore Eye Research Institute, Singapore National Eye Centre, Singapore, Singapore; 2 DUKE-NUS Graduate Medical School, Singapore, Singapore; 3 Singapore Institute for Clinical Sciences, Growth, Development & Metabolism, Singapore, Singapore; 4 Department of Nutrition and Dietetics, Flinders University of South Australia, Adelaide, South Australia, Australia; 5 Singapore Institute for Clinical Sciences, Agency for Science, Technology and Research, Singapore, Singapore; 6 Department of Paediatrics, Yong Loo Lin School of Medicine, National University of Singapore and National University Health System, Singapore, Singapore; 7 Khoo Teck Puat- National University Children’s Medical Institute, National University Health System, Singapore, Singapore; 8 KK Women’s and Children’s Hospital, Singapore, Singapore; 9 Department of Obstetrics and Gynecology, Yong Loo Lin School of Medicine, National University of Singapore, Singapore, Singapore; 10 Saw Swee Hock School of Public Health, National University of Singapore, Singapore, Singapore; 11 Medical Research Council Lifecourse Epidemiology Unit and NIHR Southampton Biomedical Research Centre, University of Southampton and University Hospital Southampton NHS Foundation Trust, Southampton, United Kingdom; Medical Clinic, University Hospital Tuebingen, GERMANY

## Abstract

**Background:**

Imbalanced macronutrient intakes can induce impairment of endothelial and vascular function, and further lead to metabolic and cardiovascular disease. However, little is known about the influence of such diets on endothelial and vascular dysfunction in pregnant women, even though high-fat diet is a known risk for pregnancy complications such as gestational diabetes and pre-eclampsia.

**Objective:**

We aimed to assess the association between maternal macronutrient intakes (protein, fat and carbohydrates), dietary quality and retinal microvascular changes in a multi-ethnic Asian mother-offspring cohort.

**Methods:**

Pregnant women (n = 614) with singleton pregnancies were recruited during their first trimester from June 2009 to Sep 2010. Maternal diet quality and macronutrient intakes, expressed as a percentage of total energy during pregnancy, were ascertained using 24 hr recalls and 3 d food diaries at 26–28 weeks gestation. Retinal examination was completed at the same clinic visit. Dietary quality was assessed and scored using the Health Eating Index in Asian Pregnant women (HEI-AP), while macronutrients intakes ware expressed as percentages of total energy and further log transformed for analysis. Associations were examined cross-sectionally by substitution models with the use of multiple linear regression.

**Results:**

In adjusted model, each 20 points decrease in HEI-AP score was associated with a significant increase of 1.70 μm (p<0.05) in retinal venular calibre. Each 0.1 log increase in percentage of total fat intake was associated with a significant increment of 1.84 μm (p<0.05) in retinal venular caliber. Additionally, each 0.1 log increase in percentage of mono-unsaturated fat intake was associated with an increment of 1.84 μm (p<0.01) in retinal venular caliber.

**Conclusions:**

In this cross-sectional study, we found that women with higher fat and lower protein intakes, and lower diet quality tended to have wider retinal venular caliber, which is suggestive of suboptimal microvasculature.

## Introduction

Macronutrients, i.e. protein, fat and carbohydrates, supply energy to meet the body’s need for specific physiological function [1,2]. The imbalance in the proportions of macronutrient intakes such as a high-fat diet has been shown to be associated with long-term increased risk of metabolic disorders [3–5] and cardiovascular diseases [6–8]. Substantial evidence from animal experiments [9–11] and human studies [12–15] has suggested that impairment of endothelial and vascular function, resulting from either high-fat diets or advanced glycation end products, could be the major underlying mechanism. In recent decades, dietary macronutrient intakes have also been found to be associated with pregnancy complications such as gestational diabetes [16–19] and pre-eclampsia [20–22], possibly via the pathways of endothelial dysfunction, inflammatory activation and oxidative stress [22,23]. Whether such diets during pregnancy also impact on the microvasculature, a surrogate marker of endothelial and vessel function *in vivo*, is unknown.

With the help of recent technological advancement, microvasculature *in vivo* has provided a valuable “window” through which clinicians are able to monitor endothelial function and inflammation in human subjects in a non-invasive manner [24]. A series of retinal vascular abnormalities (e.g. retinal arteriolar narrowing, retinal venular widening, higher length-to-diameter ratio and more tortuous retinal arterioles) have been repeatedly proven to be associated with biomarkers linked to endothelial dysfunction, inflammation and dyslipdemia [25–29]. In adults and children, studies have shown that suboptimal dietary intakes such as high sugar, low fibre and low fish oil intakes were associated with retinal vascular changes (e.g. narrower retinal arterioles, wider retinal venules and lower fractal dimension), which are consistent to the abnormalities mentioned above [30–33].

Therefore, we aimed to study the association between maternal macronutrient intakes and maternal retinal microvasculature in a cohort of 614 pregnant women. We hypothesized that pregnant women with high-fat diet and poorer diet quality represented by lower dietary quality indexes were more susceptible to microvascular abnormality such as retinal arteriolar narrowing or retinal venular widening.

## Methods

### Study population and design

This is a hospital-based, cross-sectional observational study. Women with singleton pregnancies were recruited during their first trimester in the on-going birth cohort study, the Growing Up in Singapore Towards Healthy Outcomes (GUSTO), from June 2009 to Sep 2010. Pregnant women who were Singaporean residents aged 18 years and above, attending either KK Women’s and Children’s hospital (KKH) or National University Hospital (NUH), and intending to deliver and reside in Singapore for the next 5 years were included. The details of the study methodology have been reported elsewhere [34].

Among 1163 women recruited in this cohort, 614 subjects completed both retinal photography and food dietary questionnaire during mid-late gestation (26–28 weeks’ pregnancy). The reason of the subset being chosen was due to the logistic issue that only participants from KKH were able to get access to retinal examination.

### Ethics Statement

This study was approved by both SingHealth Centralized Institutional Review Board and the National Health Group’s Domain Specific Review Board, and it was conducted according to the tenets of the Declaration of Helsinki. Written informed consent in 3 copies were obtained from participants prior to any examination. Among these 3 copies, 1 was kept by the mothers, 1 was for the study principal investigator and another was kept at the research site. SingHealth Centralized Institutional Review Board had approved such consent procedure before this study was conducted.

### Dietary Assessment

#### Macronutrients Assessment

Dietary intakes were ascertained with the use of a 24 hour recall during the 26–28 week’ gestation visit [35–37]. A 5-stage, multiple-pass interviewing technique was applied in the 24 hour recall questionnaire conducted by trained clinical staff [38]. To assist women in quantifying their food and beverage intake, visual aids such as standardized household measuring utensils and food pictures of various portion sizes were presented. All subjects were familiarized with the food pictures and then were led through the interview by the trained interviewer. The multiple-pass assessment consists of 5 steps: 1) the quick list, which is an uninterrupted listing by the subjects of foods and beverages consumed; 2) the forgotten foods list, which queries the subject on categories of foods that have been documented as frequently forgotten; 3) a time and occasion at which foods were consumed; 4) the detailed cycle, which elicits descriptions of foods and amounts eaten aided by the interactive use of the food pictures and measuring guides; and 5) the final probe review. The women were also asked to complete a 3-day food diary at home, following the visit. These were returned to the clinic staff at the following visit. Only a subset of women (n = 835) completed and returned the diaries. Dietary data from the 3-day food diaries was used to validate the dietary data obtained from the 24 hr recalls. Results from 24 hour recalls relating to health outcomes and those from food diaries relating to similar health outcomes have been shown to be comparable [36]. Such methods of assessing dietary data have been widely published and accepted [39–41].

Food diaries were returned at the next clinic visit and analyses of the dietary records were performed with the use of nutrient analysis software (Dietplan, Forestfield Sofrware, U.K.), or the dietary records composition database derived on locally available foods [36,42]. Energy-adjusted macronutrient intakes were expressed as percentage of total energy, such as percentage fat of total energy, percentage carbohydrates of total energy etc. Different fat types such as saturated fat, mono-unsaturated fat (MUFA) and poly-unsaturated fat intakes (PUFA) were also expressed as % of total energy. The macronutrient and fat types analyses in this study were based on data from the 24 hour recalls.

#### Diet quality

is measured by scoring food patterns in terms of how closely they align with national dietary guidelines and how diverse the variety of healthy choices is within core food groups or equivalent international groupings [43]. The healthy eating index for Asian pregnant women (HEI-AP) was developed and validated locally to examine dietary quality in pregnant women [44]. It was adapted from both the Healthy Eating Indices (HEI) [45–47] and Alternative Healthy Eating Index for Pregnancy (AHEI-P) [48] and modified accordingly to recommendations from the Singapore dietary guidelines for pregnant women [49]. In brief, the HEI-AP consists of 11 components including total fruit, whole fruit, total vegetables, dark green leafy and orange vegetables, total rice and alternatives, whole grains, dairy, total protein foods, use of antenatal supplements, total fat and saturated fat. Out of the 11 components, there are 8 food-based components (4-adequacy of food groups, 4–quality of food groups), 2 nutrient-based components to reflect nutrients to be taken in moderation, and 1 to assess adherence to antenatal supplements. Minimum and maximum scores range between 0 and 5 points for the first four components, and range between 0 and 10 points for the rest of the seven components. The total raw score is up to 90 and scaled up to 100 as per the referenced HEI [44] (S1 Fig). By using a density basis of recommended serve size per 1000kcal, each individual’s reported energy intake was adjusted for energy intake and the diet quality score was more comparable between individuals [44]. For each woman, a diet quality score is calculated. The higher the score, the closer adherence the diet is to guidelines and thus reflects better quality of the diet. Further details of the HEI- AP can be found in Han et al, 2015 [44].

### Retinal Photography and Measurements of Retinal Vascular Parameters

**Fig 1:**
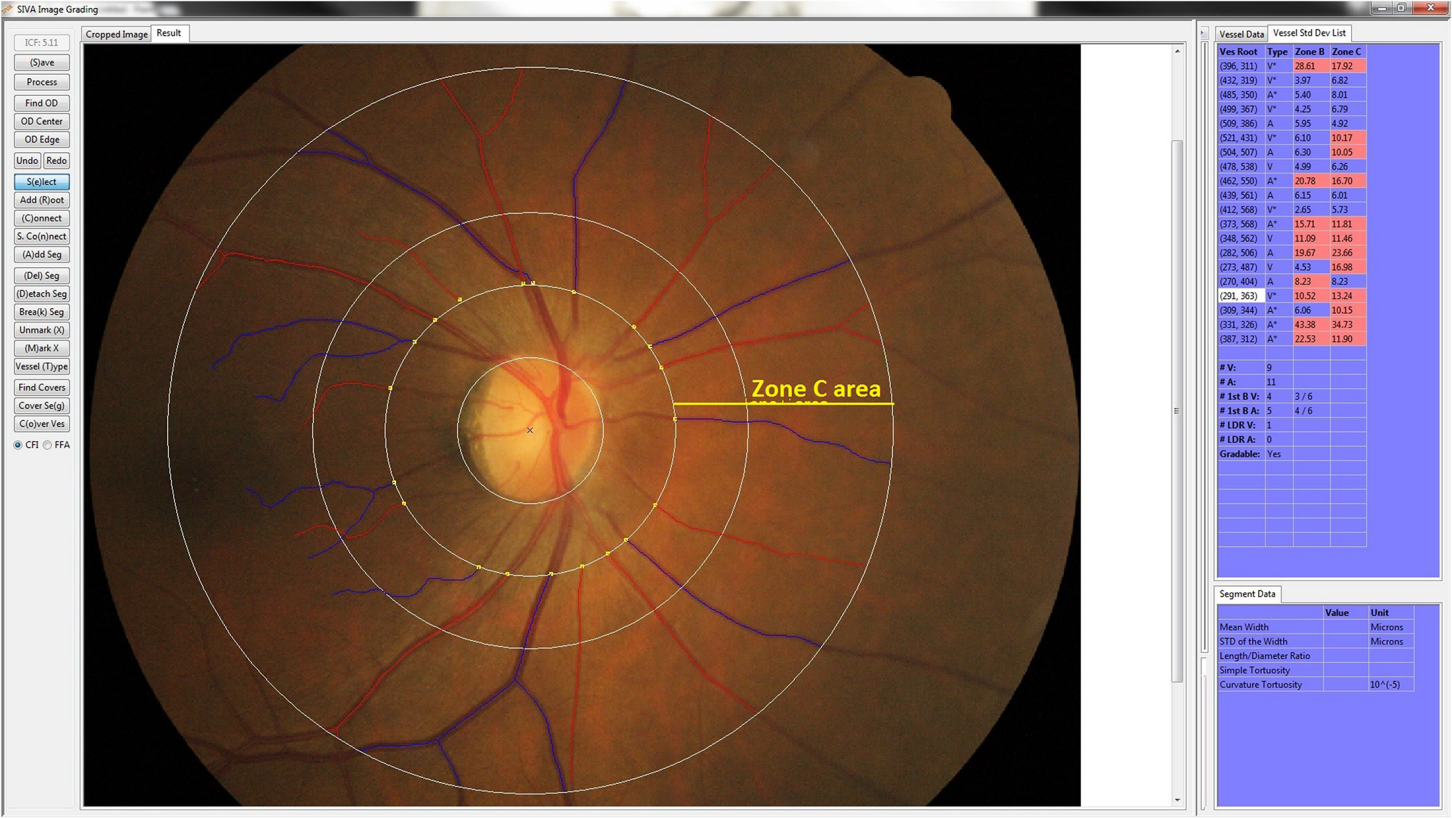
Image of SIVA grading platform. Retinal microvasculature assessment on the grading platform. A screenshot of a computer-assisted program for measurement of new geometrical retinal vascular parameters from retinal fundus photograph. Zone C is marked in SIVA software by 0.5 to 2.0 optic disc diameter away from the margin of optic disc, respectively. All retinal arterioles and venules larger than 25 μm are marked and assessed within zone C.

**Fig 2:**
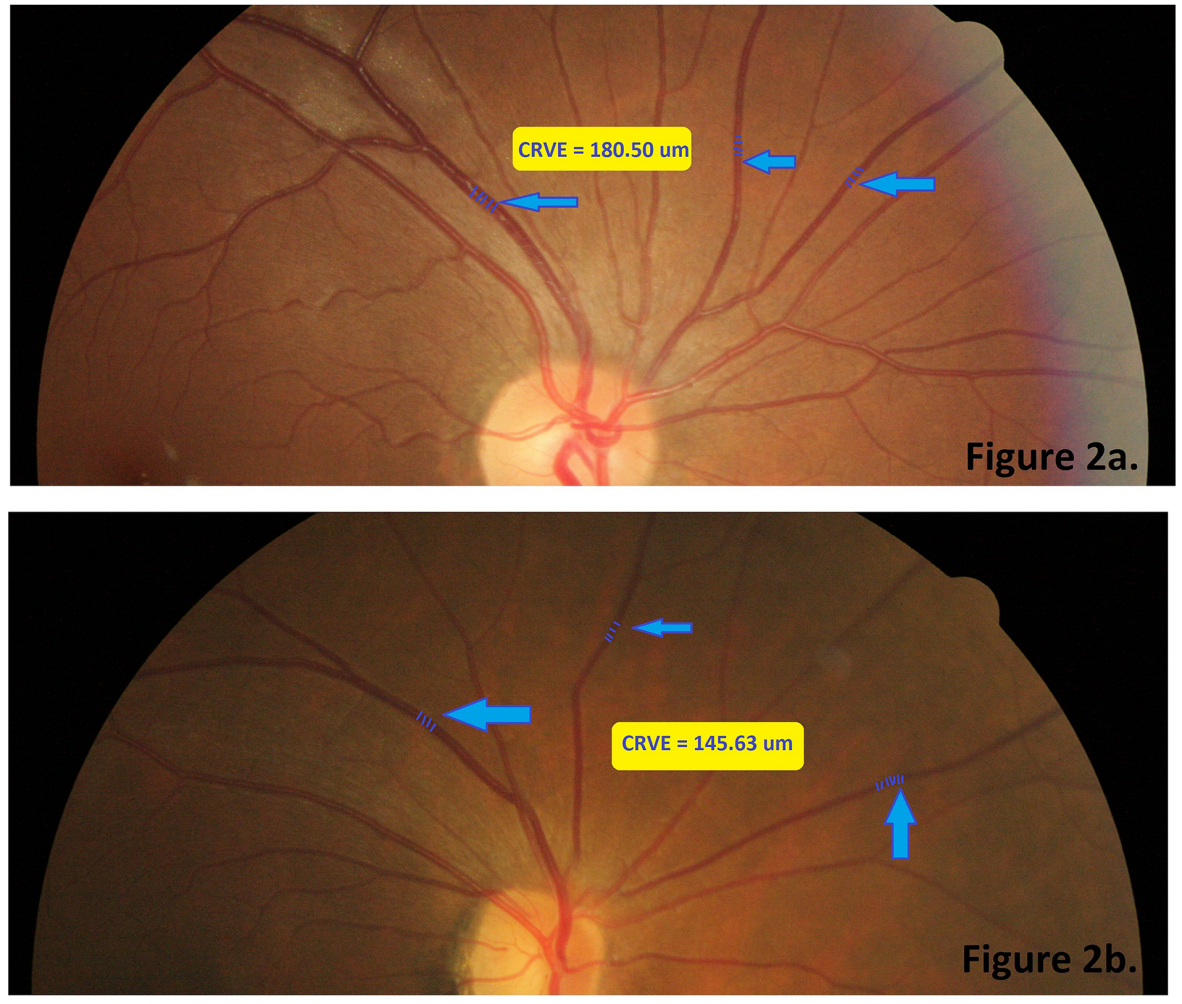
Examples of retinal venular assessment from two subjects with different diet quality. Subject in Fig 2a in the upper panel has a unhealthier diet pattern than the subject in Fig 2b. in the lower panel. Comparisons between 2a and 2b are listed below: total mono-unsaturated fat (MUFA) percentage—80.25% vs. 13.45%; total fat intake percentage– 41.8% vs. 36.9%; Health Eating Index for Asian Pregnant Women (HEI-AP)– 27.20 vs. 38.70; central retinal venular equivalent (CRVE)– 180.50 μm vs 145.63 μm.

Retinal photography and measurements of retinal vascular parameters were performed at the same clinic visit where 24 hr food recall data were collected. Right eye retinal photographs were performed without pharmacological pupil dilation using a 45° non-mydriatic retinal camera (Canon CR-1, 40D SLR digital-retinal camera backing, Canon Inc., Japan). All retinal photographs were centered on the optic disc and then assessed by a trained grader using a semi-automated computer-based program (Singapore I Vessel Assessment [SIVA] version 3.0, Singapore Eye Research Institute, Singapore). All vessels located in zone C which was marked in SIVA software by 0.5–2.0 optic disc diameter away from the optic disc margin were recruited for calibration (Fig 1). Retinal vascular caliber, represented as central retinal arteriolar equivalent (CRAE) and central retinal venular equivalent (CRVE), was assessed. Morphological differences between normal and narrow CRVE among our subjects are shown in Fig 2.

The gradability of all the mothers’ retinal photographs were up to 98% as reported in previous publications for the same cohort [50,51]. Intra-grader reliability, which was assessed in a ~10% subset (n = 60) with randomly selected retinal photographs from same cohort, ranged from 0.95 to 0.99 for retinal vascular caliber readings.

### Assessment of Co-Variates

#### Blood Pressure Measurement

Left upper arm blood pressure was measured using the automatic Omron sphygmomanometer (Omron HEM 705 LP, Omron Healthcare Inc., US) with an upright seating posture after at least 5 minutes of rest [50,51]. Only two readings were required. Unless the difference was greater than 10 mmHg in systolic blood pressure (SBP) and/or 5 mmHg in diastolic blood pressure (DBP), respectively, a third reading would be applied to calculate the average of the 2 closest readings of SBP and DBP. Mean arterial blood pressure (MABP) was calculated as one third of the SBP plus two thirds of the DBP.

#### Anthropometric measurement

Height and weight were both measured with standing posture using standard protocol of SECA weighing scale (Vogel and Halke, Germany) [50,51]. Two readings were required with bare feet. Unless the difference was greater than 1.0 cm or 200 grams for height or weight, a third reading would be required for the average height and weight calculation. Body mass index (BMI) was calculated as weight divided by the height squared (kilograms [Kg] per meter squared [m^2^]).

#### Other Covariates

Maternal demographic status (maternal education and household income), personal medical history (hypertension and diabetes), smoking, alcohol intake, parity and pre-pregnancy weight were all ascertained using questionnaires administered by trained clinic staff.

### Statistical Analysis

Based on the normal distribution of retinal vascular caliber, HEI-AP and total energy intake, all these variables were analyzed as continuous variables. However, the distribution of macronutrients (expressed as % of total energy) and fat types were skewed either to the left or to the right, and were thus log transformed for normalization, before analyses as continuous variables. Comparisons of basic characteristics between included and excluded subjects were done either by student’s t-test or χ^2^ test. P trend of categories in major variables in relation to retinal vascular caliber was analyzed by factorregression.

Multiple linear regression models were constructed to assess the cross-sectional association between maternal macronutrients and retinal vascular caliber. Stepwise backward methods were selected to choose the most parsimonious and best fitting model. A few models were applied in all analyses. Model 1, unadjusted; Model 2, adjusted for age and ethnicity; Model 3, adjusted for covariates in Model 2 and further adjusted for history of hypertension, history of diabetes, pre-pregnancy overweight or obese status, weight gain at 26–28 weeks and primiparous status.

To evaluate the effects of one macronutrient relative to another in iso-caloric diets, substitution models were further applied in Models 4 to 9 [36,52]. The purpose of applying substitution models was to account for the increment of a macronutrient at the expense of another while keeping the total energy intake constant. For example, when fat and total energy intakes are kept constant, the only macronutrient that can decrease as the percentage of energy from carbohydrate increases is protein. Thus, this model can be considered to be the theoretical substitution of carbohydrates for protein.

Besides covariates in Model 3, model 4 was additionally adjusted for total energy intake and percentage of total fat, to examine the effect of a higher carbohydrate and lower iso-caloric protein diet on retinal vascularisation. In Model 5, co-variates in model 3 were included and then additionally adjusted for total energy intake and percentage of total carbohydrate, to examine the effect of a higher fat, lower protein diet on retinal vascularisation. In Model 6, co-variates in model 3 were included and then additionally adjusted for total energy intake and percentage of total protein, to examine the effect of a higher fat, lower carbohydrate diet on retinal vascularisation.

Similarly, the substitution models were applied for the analyses of different fat types including saturated fat, mono-unsaturated fat (MUFA) and poly-unsaturated fat (PUFA) on retinal vascularisation. Three additional models were applied as below: In Model 7, co-variates in model 3 were included and then additionally adjusted for total energy intake, percentage of total protein, percentage of total carbohydrate and saturated fat percentage, to examine the effect of a higher MUFA, lower PUFA diet or vice versa. Model 8 was adjusted for percentage of MUFA to examine the effects of a higher saturated, lower PUFA diet and vice versa and finally model 9 to examine the effects of a higher saturated, lower MUFA or vice-versa.

A significant p value (2-tailed) was defined as <0.05. All statistical analyses were performed using PASW 19.0 (SPSS Inc, Chicago, U.S.). Effect modification and interaction were tested.

## Results

Comparisons of basic characteristics between included and excluded participants are shown in Table 1. Mothers included in this study were similar to those excluded except for a slightly younger age (30.49 years vs. 31.29 years), lower education level (24.27% vs. 39.05% with university degree, being more likely to smoke (13.84% vs. 11.15%) and less likely to drink alcohol (27.85% vs. 37.48%). The mean and standard deviation (SD) of retinal vascular caliber is 120.89 μm (SD = 9.01 μm) in arterioles and 171.33 μm (SD = 12.54 μm) in venules, respectively.

**Table 1:** Comparison of baseline characteristics between participants included and excluded in the study.

Characteristics	Participants		p value*
	attended	not attended	
	(n = 614)	(n = 549)	
	(mean, SD)	(mean, SD)	
Age, years	30.49, 5.45	31.29, 4.86	**<0.01**
Ethnicity, Chinese	49.59	58.39	**<0.001**
Maternal Education, University	24.27	39.05	**<0.001**
Hypertension history, Yes %	1.96%	2.55%	0.61
Diabetes history, Yes %	0.98%	2.01%	0.27
weight before pregnancy, Kg	56.93, 11.95	56.89, 11.16	0.90
BMI at 26–28 weeks’ pregnancy, Kg/m^2^	26.32, 4.49	26.00, 4.86	0.26
Primiparous, Yes %	37.91%	40.23%	0.43
CRAE, μm	120.89, 9.01	120.47, 8.30	0.58
CRVE, μm	171.33, 12.54	169.95, 12.69	0.21

*Statistical analysis was performed either by student’s t-test or χ^2^ test.

In Table 2, major variables associated with our retinal vascular caliber outcomes were categorized by clinical cut-offs or by 50th percentile. Traditional risk factors such as older age, race with darker skin pigmentation, history of hypertension and diabetes were all correlated with different retinal vascular morphological changes. Interestingly, we also found that pre-pregnancy overweight or obese status, higher weight gain during mid-late pregnancy and being primiparous were correlated with either narrower retinal arteriolar caliber or wider retinal venular caliber.

**Table 2:** Basic characteristic in categories in relation to retinal vascular caliber in the GUSTO cohort.

Variables	CRAE, μm	p trend	CRVE, μm	p trend
	mean, SD		mean, SD	
**Demographics**				
Age				
< 35 years (n = 461)	121.24, 8.88	0.17	172.49, 12.60	**<0.01**
≧ 35 years (n = 153)	120.08, 9.46		168.16, 12.31	
Ethnicity				
Chinese (n = 317)	120.00, 9.10	**<0.01**	169.21, 12.39	0.08
Malay (n = 187)	121.59, 8.98		175.00, 12.86	
Indian (n = 110)	122.63, 8.66		171.64, 11.71	
History of hypertension				
Yes (n = 12)	114.72, 11.97	**<0.05**	166.40, 17.40	0.17
No (n = 602)	121.08, 8.93		171.51, 12.54	
History of Type 2 Diabetes				
Yes (n = 6)	123.65, 6.73	0.46	171.35, 12.67	0.25
No (n = 608)	120.93, 9.05		171.33, 11.01	
**Anthropometrics**				
Pre-pregnancy overweight or obese status				
Yes ≧ 25.00 kg/m^2^ (n = 213)	119.47, 9.00	**<0.01**	171.23, 12.87	0.97
No < 25.00 kg/m^2^ (n = 353)	121.75, 8.92		171.27, 12.64	
weight gain, mean = 8.45 kg				
≤ 50 centile (n = 267)	120.80, 8.79	0.89	169.82, 12.40	**<0.05**
> 50 centile (n = 282)	120.91, 9.12		172.29, 12.82	
**Delivery data**				
Parity				
Primiparous (n = 253)	121.86, 8.62	**<0.05**	173.77, 12.42	**<0.001**
at least 1 live birth history (n = 357)	120.28, 9.27		169.65, 12.50	

P trend was calculated by factor regression.

Abbreviation: CRAE, central retinal arteriolar equivalent; CRVE, central retinal venular equivalent.

Table 3 shows the associations of dietary quality (HEI-AP) and total energy intake with retinal vascular caliber. No associations were observed between total energy intake and retinal vascular caliber. The association between poorer HEI-AP and retinal venular caliber widening was consistently significant across Models 1 to 3. In fully adjusted model, lower HEI-AP score was associated with retinal venular widening (β = 1.70 μm per 20 scores decrease in HEI-AP, p<0.05).

**Table 3:** Associations between health eating index (HEI), total energy intakes and retinal vascular caliber among pregnant women during 26–28 weeks’ gestation.

Retinal vessels	HEI-AP		Total energy intake	
	Each 20 scores ↓		Each 1.0 kcal ↑	
**CRAE, μm**	**β, SE**	**p value**	**β, SE**	**p value**
Model 1	2.02, 1.14	0.08	2.50, 2.60	0.34
Model 2	1.70, 1.12	0.13	3.66, 2.60	0.16
Model 3	1.72, 1.20	0.15	3.59, 2.76	0.19
**CRVE, μm**	**β, SE**	**p value**	**β, SE**	**p value**
Model 1	2.48, 0.80	**<0.01**	-2.23, 1.86	0.23
Model 2	1.58, 0.80	**0.05**	-1.03, 1.89	0.59
Model 3	1.70, 0.86	**<0.05**	-1.24, 1.99	0.53

Abbreviation: SE, standard error, CI, confidence interval; CRAE, central retinal arteriolar equivalent; CRVE, central retinal venular equivalent; HEI-AP, Healthy Eating Index for Asian Pregnant women.

Model 1, unadjusted

Model 2, adjusted for age and ethnicity.

Model 3, Model 2 and additionally adjusted for history of hypertension, history of diabetes, pre-pregnancy overweight or obese status, weight gain at 26–28 weeks and primiparous status.

Table 4 shows the associations between percentage of each macronutrient intake in log scale and retinal vascular caliber. In Model 5, a higher fat and lower protein diet was associated with retinal venular widening (β = 1.84 μm per 0.1 log increase in percentage of total fat intake, p<0.05). Similarly, a higher carbohydrate and lower protein diet had a borderline significant association with retinal venular widening (β = 2.17 μm per 0.1 log increase in percentage of total carbohydrates intake, p = 0.07) (Model 4). No association was seen with a higher fat and lower carbohydrate diet.

**Table 4:** Associations between Macronutrients percentage and retinal vascular caliber among pregnant women during 26–28 weeks’ gestation.

Retinal vessels	Total protein %		Total fat %		Total carbohydrates %	
	Each 0.1 unit ↑ in log		Each 0.1 unit ↑ in log		Each 0.1 unit ↑ in log	
**CRAE, μm**	**β, SE**	**p value**	**β, SE**	**p value**	**β, SE**	**p value**
Model 1	-0.47, 0.34	0.17	0.29, 0.32	0.47	0.17, 0.46	0.72
Model 2	-0.25, 0.34	0.46	0.39, 0.32	0.22	-0.14, 0.46	0.77
Model 3	-0.17, 0.35	0.64	0.25, 0.33	0.45	-0.01, 0.47	0.98
Model 4	-0.17, 0.36	0.63	--	--	0.89, 0.88	0.31
Model 5	-0.16, 0.40	0.70	0.68, 0.63	0.29	--	--
Model 6	--	--	0.15, 0.35	0.66	0.01, 0.55	0.99
**CRVE, μm**	**β, SE**	**p value**	**β, SE**	**p value**	**β, SE**	**p value**
Model 1	-0.82, 0.47	0.08	0.37, 0.44	0.41	0.43, 0.64	0.50
Model 2	-0.55, 0.47	0.24	0.51, 0.44	0.24	0.01, 0.64	0.88
Model 3	-0.57, 0.49	0.24	0.42, 0.46	0.36	0.11, 0.66	0.87
Model 4	-0.63, 0.49	0.20	--	--	2.17, 1.21	0.07
Model 5	-0.73, 0.56	0.19	1.84, 0.88	**<0.05**	--	--
Model 6	--	--	0.58, 0.48	0.23	-0.44, 0.76	0.56

Abbreviation: SE, standard error; CRAE, central retinal arteriolar equivalent; CRVE, central retinal venular equivalent.

Model 1: unadjusted

Model 2: adjusted for age and ethnicity.

Model 3: includes Model 2 and additionally adjusted for history of hypertension, history of diabetes, pre-pregnancy overweight or obese status, weight gain at 26–28 weeks and primiparous status.

Model 4: includes Model 3 and additionally adjusted for total energy intake and total fat intake percentage.

Model 5: includes Model 3 and additionally adjusted for total energy intake and total carbohydrate intake percentage.

Model 6: includes Model 3 and additionally adjusted for total energy intake and total protein intake percentage.

Table 5 shows the associations between percentage of each fat type in log scale and retinal vascular caliber. Higher MUFA and lower PUFA or saturated fats diets were consistently associated with retinal venular widening in Model 2, 3 and 7 (β = 1.84 μm per 0.1 log increase in percentage of MUFA intake, p<0.01). An example of changes in retinal venular is shown in Fig 2. Such changes in retinal venular due to different dietary pattern were shown in Fig 2.

**Table 5:** Associations between Saturated fat and retinal vascular caliber among pregnant women during 26–28 weeks’ gestation.

Retinal Vessels	Saturated Fat%,		MUFA %,		PUFA %	
	each 0.1 unit ↑ in log		each 0.1 unit ↑ in log		each 0.1 unit ↑ in log	
**CRAE, μm**	**β, SE**	**p value**	**β, SE**	**p value**	**β, SE**	**p value**
Model 1	-0.01, 0.38	0.99	0.19, 0.43	0.67	-0.08, 0.23	0.72
Model 2	-0.15, 0.39	0.69	0.43, 0.44	0.32	-0.05, 0.23	0.83
Model 3	-0.30, 0.41	0.47	0.72, 0.45	0.11	0.12, 0.25	0.64
Model 7	--	--	0.71, 0.46	0.12	-0.01, 0.38	0.80
Model 8	-0.32, 0.42	0.45	--	--	0.30, 0.27	0.26
Model 9	-0.55, 0.62	0.38	0.94, 0.54	0.08	--	--
**CRVE, μm**	**β, SE**	**p value**	**β, SE**	**p value**	**β, SE**	**p value**
Model 1	0.41, 0.54	0.44	1.01, 0.60	0.10	-0.48, 0.32	0.14
Model 2	-0.15, 0.53	0.78	1.55, 0.60	**0.01**	-0.28, 0.31	0.37
Model 3	-0.25, 0.56	0.66	1.82, 0.62	**<0.01**	-0.29, 0.35	0.41
Model 7	--	--	1.84, 0.63	**<0.01**	-0.82, 0.53	0.13
Model 8	-0.15, 5.81	0.80	--	**--**	0.07, 0.38	0.85
Model 9	-1.35,0.87	0.12	1.76, 0.75	**<0.05**	--	--

Abbreviation: MUFA, mono-unsaturated fat; PUFA, poly-unsaturated fat; SE, standard error; CRAE, central retinal arteriolar equivalent; CRVE, central retinal venular equivalent.

Model 1: unadjusted

Model 2: adjusted for age and ethnicity.

Model 3: incluing Model 2 and additionally adjusted for history of hypertension, history of diabetes, pre-pregnancy overweight or obese status, weight gain at 26–28 weeks and primiparous status.

Model 7: including Model 3 and additionally adjusted for total energy intake, total protein intake percentage, total carbohydrates percentage and total saturated fat intake percentage.

Model 8: including Model 3 and additionally adjusted for total energy intake, total protein intake percentage, total carbohydrates percentage and MUFA percentage.

Model 9: including Model 3 and additionally adjusted for total energy intake, total protein intake percentage, total carbohydrates percentage and PUFA percentage.

No associations were found between the dietary quality index or macronutrient intakes and retinal arteriolar caliber in our cohort. No evidence was found for effect modification or interaction between exposure variables in our study.

## Discussion

In our hospital-based, cross-sectional study among multi-ethnicities including Chinese, Malay and Indian pregnant women during their mid-late pregnancy, women with lower dietary quality and diets of higher fat with lower protein tended to have wider retinal venular caliber.

It has been widely accepted that high-fat diet can greatly contribute to long-term development of metabolic disorders [3–5] and cardiovascular disease [6–8]. Substantial evidence from animal experiments [9–11] and human studies [12–15] has suggested that impairment of endothelial and vascular function resulted from either high-fat diet or advanced glycation end products could be the major underlying mechanism. Recent rodent experiments showed that high fat intakes not only increased body weight, but also up-regulated the expression of hypothalamic neuropeptides which were associated with increased visceral and liver adiposity [10,53]. These two types of adiposity are both known to be detrimental to health and associated with disturbed metabolism [54,55]. One of the many hypothesized underlying mechanisms is due to endothelial dysfunction which further leads to small vessel dysfunction and ultimately results in large vessel dysfunction [10]. Thus, high-fat diet related endothelial dysfunction seems to be partially mediated by disturbed production of a wide range of adipose-derived adipokines and inflammatory factors [10,56], and thus subsequently leads to oxidative stress, a possible mediator of vascular dysfunction [10,57].

However, endothelial dysfunction is difficult to assess accurately *in vivo*, even though substantial evidence has illustrated the important role of endothelial dysfunction as an early event and an underlying factor for metabolic and cardiovascular states including atherosclerosis, hypertension and diabetes [10,57]. The ability to detect early vascular changes *in vivo* might be a key to reflect endothelial function and real-time inflammatory changes. Retinal microvasculature is the vascular bed that can be viewed non-invasively over time [24]. Morphology of retinal vasculature such as narrowing arterioles, widening in venules, sparser in fractal dimension and more tortuous vessels have been strongly suggested to be associated with endothelial dysfunction by a number of epidemiological studies [25–29]. In recent decade, retinal imaging studies applied to adults and children also found that suboptimal dietary intakes such as high sugar, low fibre and low fish oil intakes were associated with retinal vascular abnormalities as mentioned earlier [30–33]. Unfortunately, such influence of macronutrient intakes is largely lacking in pregnancy women, despite that high-fat diet has been widely established as risk factors for pregnancy complications such as gestational diabetes and pre-eclampsia [16–22].

With the aid of retinal imaging, our study is the first one to look into microvasculature with relation to dietary macronutrient intakes in a large number of Asian pregnant women. We found that a high-fat with low-protein diet was associated with wider retinal venules. As suggested by animal and human experimental studies, high-fat diet associated endothelium-dependent vasodilator dysfunction may down-regulate nitride-oxide (NO) and prostacyclin (PGI2) that are produced by endothelium cells (ECs), which ultimately lead to vascular dysfunction [15,58,59]. Therefore, retinal venular widening might be considered as one of the early signs of vascular morphology indicating the possibility of vascular dysfunction led by sub-optimal dietary pattern. In addition, we found that lower diet quality was associated with wider retinal venules. This is in line with a recent meta-analysis on 15 cohorts, where low scores on diet quality were found to be associated with a significant increment in the risk of type 2 diabetes, cardiovascular disease and all-cause mortality by 28% [60].

Based on the observations in our study, we agree with the reviewer that “low protein” with poor-diet style in terms of either high fat or high carbohydrate intake is associated with adverse retinal microvasculature. While current associations seem to suggest that lower protein diets may play a role in influencing retinal vascular caliber outcomes, the other associations examining higher protein diets at the expense of fat or carbohydrates do not appear to support this. This may in part be due to the small or tight range of log transformed dietary protein intakes percentage in our cohort and this can be further explored in future studies.

Interestingly, in the analysis of each fat component in relation to retinal vascular abnormality, higher MUFA percentage, instead of saturated fat or PUFA, was significantly associated with retinal venular widening. It has been widely suggested in clinical studies that MUFA and PUFA replacing saturated fat in the diet greatly reduced both total and low-density lipoprotein (LDL) cholesterol [61–63], which is beneficial to lower risk of cardiovascular disease development in the future. However, animal studies investigating the influence of dietary fats on atherosclerosis have reported conflicting results. Earlier work on mice and primates showed that a MUFA diet led to more atherosclerosis than both saturated fat and PUFA diets despite a more favourable plasma lipid profile [64–66]. And in our study, our findings between MUFA and retinal vascular abnormality seemed to support such observational result.

The strength of our study includes a large sample size, a novel population (pregnant women) which has not been studied on before, standardized protocols using validated assessments of retinal vasculature, detailed dietary assessments together with detailed information on a range of potential confounders. However, our study also carried a few methodological issues limiting the significance of our study. Firstly, there might be a limited role for selection bias in the 614 participants selected from a total number of 1163 subjects in the whole cohort, even though we did not find any significant differences in major baseline characteristics between these 2 groups. Secondly, recall bias and information bias might occur during only one time dietary data interview during the whole pregnancy. Thirdly, there might be residual confounders such as glycated hemoglobin, lipid profile and C-reactive protein affecting the associations investigated in this study yet not being adjusted for. Due to the lacking of such data, future studies should take these confounders into account.

In this cross-sectional study, we found that pregnant women with a higher fat with lower protein diet and lower dietary quality during mid-late pregnancy tended to have retinal venular caliber widening, a type of retinal vascular morphology indicative of sub-optimal microcirculature and correlated to future metabolic and cardiovascular risks. The relationship between the different types of fats such as MUFA and retinal vascularization needs to be further explored and confirmed.

## Supporting Information

S1 Fig Healthy Eating Index for Asian Pregnant Women (HEI-AP) components breakdown. (TIF)
