# Correction: The Impact of Macronutrients on Retinal Microvasculature among Singapore Pregnant Women during the Mid-Late Gestation

**DOI:** 10.1371/journal.pone.0165218

**Published:** 2016-10-18

**Authors:** Ling-Jun Li, Peng Guan Ong, Marjorelee T. Colega, Chad Yixian Han, Ling Wei Chen, Ryan Man Eyn Kidd, Ecosse Lamoureux, Peter Gluckman, Kenneth Kwek, Yap Seng Chong, Seang Mei Saw, Keith M. Godfrey, Tien Yin Wong, Mary Chong Foong-Fong, GUSTO study group

The affiliation for the fourth author, Chad Yixian Han, is incorrect. The correct affiliation should be: Department of Dietetics and Nutrition, Ng Teng Fong General Hospital, Jurong Health Services, Singapore, Singapore.
