# Supplementary figures and images for: The Impact of Macronutrients on Retinal Microvasculature among Singapore Pregnant Women during the Mid-Late Gestation
Source: PLoS One. 2016 Aug 10;11(8):e0160704. doi: 10.1371/journal.pone.0160704 (PMC4979959; doi:10.1371/journal.pone.0160704)

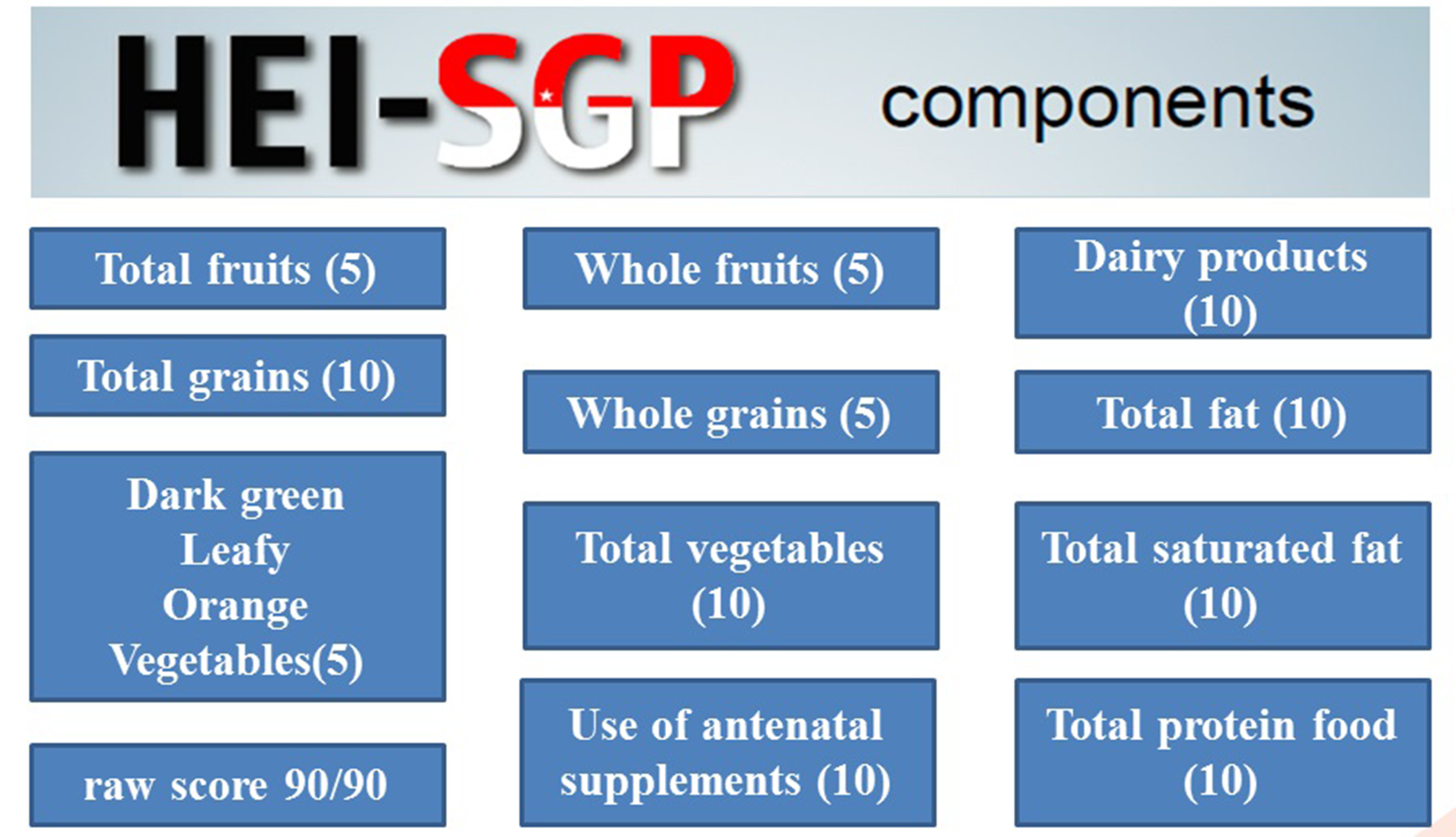

Supplement: S1 Fig — (TIF) [file pone.0160704.s001.tif]
